# Efficacy and safety of Ayurvedic medicines: Recommending equivalence trial design and proposing safety index

**DOI:** 10.4103/0974-7788.72491

**Published:** 2010

**Authors:** Sanjeev Sarmukaddam, Arvind Chopra, Girish Tillu

**Affiliations:** *Centre for Rheumatic Diseases, Hermes Elegance, 1988 Convent St, Camp, Pune – 411 008, Maharashtra, India*

**Keywords:** Efficacy, equivalence trials, safety index, safety of Ayurvedic medicines

## Abstract

Ayurvedic drugs have begun to be evaluated in controlled clinical trials. The trials, often placebo controlled, are usually designed to demonstrate superiority. Though the results have been usually reported as encouraging, statistical significance has been elusive. In this melee to show efficacy, several positive results related to safety and other purported advantages with Ayurvedic drugs, including improved quality of life, easy drug availability and less cost, get drowned. Though safety is the prime concern, efficacy ultimately matters in trials. Excellent safety profile offset modest efficacy, especially for long-term management of chronic difficult to treat disorders. There is a trade-off between efficacy and safety but we have no means to put them together in a mathematical evaluation to judge the overall performance of a drug. However, we need more suitable modern science methods/techniques to unravel the true therapeutic role of Ayurvedic drugs. We propose “equivalence trials” using modern medicine benchmark as a comparator and a “safety/tolerability index” on this perspective. We believe that several Ayurvedic drugs are capable of demonstrating equal efficacy but superior safety. Our concept may also be applicable for pragmatic trials that are more suitable for Ayurvedic therapy.

## INTRODUCTION

It is well accepted that the clinical trials are the best way to assess efficacy of treatment. However, trials in their present form may not be suitable for Complementary and Alternative Medicine (CAM).[1] Therefore, it has become essential to modify or identify methods/techniques suitable for CAM including Ayurveda.

In an effort to unravel scientific evidence using modern science means, Ayurvedic drugs have begun to be evaluated in controlled drug trials. These trials, which are often placebo controlled, are usually designed to demonstrate superiority. Though the results have been usually reported as “encouraging and merit further drug development”, the statistical significance has been elusive. In this melee to show efficacy, several positive results related to safety and other purported advantages (like improved quality of life, easy drug availability and less cost) with Ayurvedic drugs are lost or underreported. Currently used descriptive statistical methods [frequency, Confidence Interval (CI)] do not address intensity of adverse events or the intervention required to treat them. As safety is the inherent strength of Ayurvedic medicines, better safety/tolerability evaluation system is required to capture its extent. Moderate efficacy but excellent safety, which may be the case with several Ayurvedic medicines, may suffice to maintain the control in long-term management of chronic disorders such as degenerative diseases. There is a trade-off between efficacy and safety but we have no means to put them together in a mathematical evaluation to judge the overall performance of a drug.

## AYURVEDA AND TRIAL DESIGNS

The gold standard for a new drug entity in clinical research is the randomized, double-blind, placebo controlled drug trial. However, with a large number and range of medicines already available, newer medicines are increasingly being developed for indications in which a placebo control group would be unethical. Some authors have rightly debated that placebo controlled trials are unethical.[2,3] Such views would reinforce the trend toward using active comparators. In such situations, one obvious solution is to use an existing drug already licensed and regularly used as a standard of care for the indication in question as an active comparator. New treatment is then expected to match the efficacy of the standard treatment but may demonstrate other advantages in safety, convenience, or cost.

Under these circumstances, the objective of the trial is to show equivalent efficacy, the so-called “equivalence” trial. Such trials have been referred to as “active control equivalence studies” or “positive control studies” and such trials will avoid the unethical use of placebo controlled design. ICH guidelines define “equivalence trial” as “a trial with the primary objective of showing that the response to two or more treatments differs by an amount which is clinically unimportant”.[4]

## ABSENCE OF EVIDENCE AND EVIDENCE OF ABSENCE

Sometimes evidence is not sufficient to demonstrate the efficacy. But this absence of evidence does not mean evidence of absence.[5] It is probable that Ayurvedic medicines which failed to show superiority in superiority design drug trials may be equivalent to conventional medicine.

## AYURVEDA: EQUIVALENT EFFICACY AND SUPERIOR SAFETY?

Though modern medicines have often surpassed all expectations regarding efficacy, and that too often in life-threatening states, it is their toxicity profile which threatens to negate their benefits. Undoubtedly, the reductionist approach in modern medicine leads to a trade-off between efficacy and safety. Though safety is supreme, drug trials in real life are all about demonstrating efficacy. Much of significant toxicity, short of life threatening, gets buried in a highly significant *P* value (efficacy). Ideally speaking, there should be a combined and integrative approach when evaluating efficacy and safety of a drug and due weight should be assigned to safety. The latter may be more important when confronting modest efficacy in difficult to treat disorders requiring long-term management. A modest *P* value (efficacy) for a mean change does not exclude the possibility of a good response in a subset of patients, and when combined with good safety, the investigational drug merits serious consideration.

Trials could be designed with the hypothesis, “Ayurveda interventions are equivalent to conventional medicine for efficacy and superior in terms of safety”. The trade-off type matrix [Table 1] obviously presents four combinations of safety and efficacy. It is likely that in case of chronic diseases and long-term management (e.g., rheumatoid arthritis), physicians and patients may accept drugs with moderate efficacy but optimal safety. Therefore, we pose a fundamental question. How can we add another column to this matrix, with a rational acceptance of “excellent safety and moderate efficacy”?

**Table 1:** Safety–efficacy trade-off matrix

	Effective	Not effective
Safe	Safe and effective	Safe and not effective
Not safe	Not safe but effective	Not safe and not effective

## EQUIVALENCE TRIALS

If we accept that Ayurvedic medicines are likely to be safer, economical and easy to access and use, then efficacy wise an “equivalence trial” may suffice. The conventional argument in support of “equivalence trials” goes like this:[6]

In conventional superiority drug trial design, the null hypothesis (H_o_) states that both the treatments have no difference, whereas the alternate hypothesis (H_1_) states that they are not equal.

H_o_: effect of Treatment A = effect of Treatment B, i.e., H_o_: A = B and H_1_: A is not equal to B (two-sided H_1_) or A > B or A < B (one-sided H_1_), where “A” is some Ayurvedic drug and “B” standard drug (modern active comparator) available/used currently for this same disorder.

This H_o_ is tested (by appropriate test). If we do not reject H_o_ (i.e., test statistic is not significant at the given alpha level), we say that “evidence is not enough to prove A = B”. This is “lack of evidence” of equivality. However, “absence of evidence is not evidence of absence”. Therefore, if we intend to prove “equivality”, the answer is “Equivalence Trial” as the aim of equivalence trial is to show the therapeutic equivalence of two treatments.

**Figure 1:**
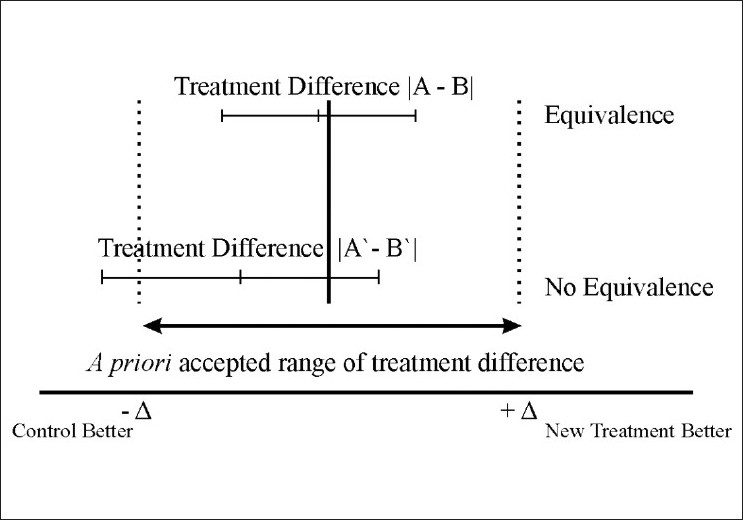
Clinical approach for analysis of equivalence trials

Quite often it is seen in the literature that the authors conclude “equivalence” after a nonsignificant result (‘negative trial’) is found in a trial that was actually designed to demonstrate real difference. It is important to note that nonsignificance does not mean equivalence. An equivalence trial is designed to confirm the absence of a meaningful difference between treatments. Though the absolute equivalence can never be demonstrated, it is possible to assert that the true difference is unlikely to be outside the “equivalence” range. In this case, it is more informative to conduct the analysis by computing the CI of the difference between the two treatments although there are closely related methods, using significance test procedures. A margin of clinical equivalence is chosen by defining the largest difference that is clinically acceptable, so that a difference bigger than this would matter in practice. If we have a predefined range of equivalence as an interval from –∆ to +∆, we can then simply check whether the CI centered on the observed difference lies entirely between –∆ and +. If it does, equivalence is demonstrated;[7] if it does not, there is still room for doubt. Possible results of the comparison of a CI with a predefined range of equivalence are shown in Figure 1.

Any CI which does not include zero corresponds to a statistically significant difference. In equivalence testing, the relevant null hypothesis is that a difference of at least ∆ exists, and the trial is targeted at disproving this in favor of the alternative that no difference exists (i.e., the difference is clinically unimportant). This formulation is important in validating the intuitive CI procedure, and it also helps in calculating sample sizes. Claim for equivalence must be based in an equivalence design and not inferred after a negative result of a trial designed to show difference.[8]

Values need to be specified for the range of equivalence (∆) and the probabilities of type I and II errors [α (alpha), and β (beta), respectively]. The choice of ∆ is difficult and requires extensive debate with knowledgeable clinical experts. The selection of α and follows similar lines as for comparative trials. The use of a 95% CI in an equivalence trial, as recommended by the European Committee for Proprietary Medicinal Products (FDA also approves this design) in its note for guidance on biostatistics, corresponds to a value for α of 0.025. However, is treated identically, and is generally set to 0.1 (to give a power of 90%) or 0.2 (to give a power of 80%). The distinction between one-sided and two-sided tests of statistical significance also carries over into the CI approach. For a one-sided test, equivalence is declared if the lower one-sided confidence limit exceeds –∆. This approach is indicated when the objective is to ensure that the new agent is not inferior to the standard and then the trial is called “non-inferiority”. E9[4] Guideline of ICH defines “non-inferiority trial” as “a trial with the primary objective of showing that the response to the investigational product is not clinically inferior to a comparative agent”. It also highlights the fact that there are well-known difficulties associated with the use of the active control equivalence (or non-inferiority) trials that do not incorporate a placebo. These relate to the implicit lack of any measure of internal validity (in contrast to superiority trials), thus making external validation necessary. Moreover, active comparators should be chosen with care. An example of a suitable active comparator would be a widely used therapy whose efficacy in the relevant indication has been clearly established and quantified in well-designed and well-documented superiority trial(s) and which can be reliably expected to exhibit similar efficacy in the contemplated active control trial. To this end, the new trial should have the same important design features (primary variables, the dose of the active comparator, eligibility criteria, etc.) as the previously conducted superiority trials in which the active comparator clearly demonstrated clinically relevant efficacy, taking into account advances in medical or statistical practice relevant to the new trial. It is prudent to add that in equivalence (or non-inferiority) trials, the comparator should be a well-accepted standard of care; otherwise, the conclusion may be confounded by “assay sensitivity”. This important issue is discussed at length in the ICH’s E10.[9]

The most common approaches to the analysis of randomized trials are “intention to treat (ITT)” and “per protocol (PP)” analyses. In an ITT analysis, patients are analyzed according to their randomized treatment, whereas PP analysis compares patients according to the treatment actually received and includes only those patients who satisfied the entry criteria and properly followed the protocol. In an equivalence trial, it is probably best to carry out both the types of analysis and hope to show equivalence in either case. ICH’s E9[4] states that in superiority trials, ITT is used in the primary analysis; but in equivalence or non-inferiority trial, use of ITT is generally not conservative. Subjects who withdraw or drop out of the treatment group or the comparator group will tend to have lack of response, and hence the results of using ITT may be biased toward demonstrating equivalence.[4] With respect to other aspects of design (like double blinding of medication, randomization) and analysis, equivalence trials are similar in nature to comparative trials. More details of equivalence trials can be found in literature.[10–12]

The utility of this philosophy was demonstrated in one of the randomized, double-blind, multicenter equivalent design drug trials conducted under a sponsorship by the Council of Scientific and Industrial Research, Government of India, New millennium Indian technology leadership initiative (NMITLI) Arthritis project. We have reported a therapeutic equivalence between standardized Ayurvedic drugs, celecoxib and glucosamine, to treat symptomatic osteoarthritis of the knees. This trial also showed good safety profile for the Ayurvedic drugs.

There could be more than one active comparator and new drug formulations to be tested in one trial which was the case in the NMITLI drug trial cited above. In any case, analysis should be based on “CIs” and this also carries implications for the estimation of the required number of patients at the design stage.

### Pragmatic trials and “black-box” design

A case is made for the appropriate use and relevance of pragmatic trials in the evaluation of alternative and complementary medicine in the article by Hugh MacPheron.[13] The main strength of pragmatic trials is that they can evaluate a therapy as it is used in normal practice. Pragmatic trial could be used to test an overall “package” of care (similar to WHO’s “black-box” design) and it is easier to grant the practitioners the freedom to treat the patients normally, allowing them to use individual approaches for different patients. It may be specifically noted that pragmatic trial philosophy goes well with the equivalence trial.

In short, the pragmatic trial concept is useful in view of the complexity of Ayurveda intervention. For such concept, trial could be equivalence and this fact is just pointed out here.

## PROPOSED “SAFETY INDEX”

Equivalence generally pertains to efficacy but it would be desirable to match the safety and tolerability of the investigational drug with that of the comparator. Often, occurrences of side effects are described as frequencies or percentages without producing exact (binomial) CIs. Overlapping of such CI in different groups can be used for “significance” inference but that will be limited to only one side effect at a time, and therefore, when equivalence design is used, subsequent statistical testing/comparison with respect to overall safety becomes essential. Also, it is difficult to measure the quantum of side effects/adverse events/toxicity in a drug trial; the abbreviation AE in the text represents any of these three events which are not strictly synonymous. In drug trials, AE can occur at any time and can be recurrent. But AEs are usually recorded at the time of predetermined time end points during the post randomization follow-up phase. Only if we could assign a numerical value to each side effect to reflect its intensity, impact and outcome, the impact in this case may be considered proportional to the remedial intervention required to treat the AE. Outcome is usually complete resolution or infrequently disability and death. One could load the “attributability” of the AE to the concerned drug to its safety profile. In a drug trial setting, it may be prudent to attribute all AE to the drug and or trial, unless proved otherwise. The latter may result in plenty of signal noise but could be sorted out to some extent by recording commonly occurring symptoms (e.g., headache, dyspeptic complaints, itching) at baseline that is likely to confound the interpretation of the AE. Of course, the control intervention arm ensures that discrimination is based on truly investigator drug related AE.

We propose the use of a “safety index” to capture the burden of AE at any time point in a trial subject. A stepwise calculation procedure of the index is described below.

*Step 1:* Fix numerical “safety value” of each AE according to categories “A” and “B”.

Category A: Severity will be a combined function of intensity and duration and will be classified as in Table 2.

**Table 2:** Scores for severity of AE

Score	Severity of AE
1	Mild
2	Moderate
3	Severe

Category B: Four grades of clinical seriousness of the AE (purposely reverse coded) are as in Table 3.

**Table 3:** Scores for severity of AE

Score	Description of clinical seriousness of AE
4	Life threatening and requiring emergency measures in a hospital
3	Inpatient (hospitalization) care lasting >24 hours An invasive procedure that confirms a tissue damage diagnosis (e.g., pepticulcer) and/or used for a specific treatment modality (e.g., block variceal bleed) Any form of parental therapy given for >3 days An AE that resolves only on stopping the interventional drug
2	Outpatient drug therapy and includes <24 hours observation in a day care facility
1	Adverse events self-managed with change in diet, lifestyle, reassurance and not requiring any of the above interventions

*Step 2:* Calculate the safety value (A × B) for each AE. The safety value of each AE will be added to calculate the “safety total” for that subject. If the AE recurs during the trial, each occurrence will be counted separately and may have a different safety value. To facilitate calculation of (A × B), the matrix form tabular display of coefficients given in Table 4 may become handy.

**Table 4:** Result of A × B in matrix form

	Category’s numeric value ↓ of B and → of A	Severity of that side effect in subject (A)		
		1	2	3
Seriousness of side effect (B)	1	1	2	3
	2	2	4	6
	3	3	6	9
	4	4	8	12

Statistical analysis: The process of assigning numerical values for “A” and “B” makes the index subjective. Nevertheless, it is likely to yield ordinal level measurements and the index is to be useful for comparison.

*Step 3:* Prepare frequency distribution of individual safety totals in a group. Arrange all frequencies in ordinal categories/classes so that relative to identified distribution (RIDIT) analysis could be used to compare groups for statistical significance.

*Step 4:* Apply RIDIT analysis to compare groups for statistical significance. The mean RIDIT yielded by this technique for a group is the probability that a randomly selected individual from it has a greater index value than a randomly selected individual from the standard group.

Similar type of index for the assessment of “socioeconomic status” based on parity dollars was suggested[14] and proved to be very useful. However, it may be noted that index’s absolute value may or may not have proper “interpretation”. Appropriateness of statistical technique to be used in this situation is amply apparent from the original paper[15] on RIDIT analysis, which also describes many real examples. Technical details and application of RIDIT analysis can also be found in literature.[10,16]

## LIMITATIONS OF PROPOSED “SAFETY INDEX”

Ayurveda is known to produce side effects which are positive, for example, most Ayurvedic treatments/therapies actually improve the functioning of “digestive system” as side effect (if we call such positive effect as side effect). Such positive side effects are not given any consideration in the construction of this index. This safety index is limited only to consider “negative” side effects.

## EPILOGUE

The “safety index” is being proposed based on the premise that Ayurvedic medicines are much gentler and require less aggressive remedial interventions. The challenge for the safety index is to capture these gentle AEs while recording the somewhat more aggressive AE with the modern medicine. Undoubtedly, we have no current tool to capture the burden of AE/toxicity with an intervention. Though empirical, safety index may make comparison between interventions for AE more robust. Establishing “criterion validity” for the index may be difficult if not impossible because there are no “gold standards” to compare with (to serve as criterion). However, there is enough “content validity”, “face validity” and “consensual validity” to this “safety index”. Since “safety” is of paramount importance and an inherent strength of Ayurvedic treatment, such an index needs to be explored in real life drug evaluation system.

## CONCLUSION

We have presented two relatively new tools to favor a suitable evaluation of Ayurvedic drugs and management. Though well described, the “equivalence trial” for efficacy has been neglected over the years but may well be the way forward with CAM, Ayurveda in particular. Our proposed “safety index” is based on an unmet need to integrate the intensity and seriousness (based on management) of an AE/drug toxicity into a single quantity and further capture mild side effects. The index should appeal to a clinical mind. It is still a concept to prove the hypothesis of “superior safety” with Ayurvedic medicines. It is prudent to add that the index is likely to find favor with the modern medicines as well.

Data from our earlier drug trials also support our basic contention in the current paper that we need better ways to evaluate Ayurvedic medicines and compare them with modern medicine.[17,18] Though popularly used as a complementary system in general medical practice, Ayurveda is often used as a truly alternative (to modern medicine) medicinal system in several parts of India (especially in the state of Kerala). Doctors in India often prescribe Ayurvedic and modern medicines together though the scientific evidence of such integration is still lacking. Undoubtedly, there is a need to set up a modern medicine–Ayurveda interface to define newer and better therapeutic strategies. The latter is likely to yield greater success in difficult to treat chronic ailments such as arthritis.

**Key points for equivalence trials**

1. Use comparators whose superiority over placebo is established.
2. Range of equivalence should be well defined clinically, before starting equivalence trial (i.e. in Protocol)
3. All issues including double blinding of medication and randomization are equally important as in comparative trials

**Further reading**

1. Jones B, Jarvis P, Lewis JA and Ebbutt AF: Trials to assess equivalence : the importance of rigorous methods, BMJ 1996; 313:36-39
2. ICH’s E9: Statistical Principles for Clinical Trials
3. ICH’s E10: Choice of control group and related issues in clinical trials

**What this article adds**

1. A strategy for evidence for Ayurvedic treatment should be thought on the background of the demand for Evidence Based Medicine
2. We hypothesize that Ayurvedic treatment could be demonstrated having ‘equivalent efficacy – but superior safety’ in comparison with modern medicine

AE will be classified as mild, moderate or severe as per physician’s discretion. Each AE will be classified after observing its course from onset till resolution or end of the study or earlier if prematurely withdrawn and not followed. Any AE lasting for more than a week will be moderate or severe

The above pertains to a grading system based on the clinical “seriousness” of management of the AE and not its grade of severity. In case of a residual disability/loss of vital function following an AE, the score will be increased by 1 in case of a mild outcome and by 2 if the outcome is functionally significant. Therefore, the maximum score in category B will be 6
